# Physician-facilitated designation of proxy decision-makers: family physician perceptions

**DOI:** 10.1186/s13584-016-0059-6

**Published:** 2016-04-30

**Authors:** Gideon Lifshitz, Matan J. Cohen, Hila Shmilovitz, Mayer Brezis, Amnon Lahad, Arie Ben-Yehuda

**Affiliations:** Clalit Health Services, Jerusalem district, Jerusalem, Israel; Department of Medicine, Hadassah-Hebrew University Medical Center, Jerusalem, Israel; Center for Quality and Clinical Safety, Hadassah-Hebrew University Medical Center, Jerusalem, Israel; Department of Family Medicine, Hadassah-Hebrew University Medical Center, Jerusalem, Israel

**Keywords:** End-of-life care, Proxy decision-makers, Family physicians

## Abstract

**Background:**

Among the challenges encountered during the care of patients at the end-of-life (EOL), eliciting preferences of patients with whom there is no ability to communicate is common and stressful for all those concerned and charged with patient care. Legal facilities available include patient delegation of proxy decision-makers (PDM) prior to communication incapacity. We sought to estimate family physician awareness and attitude with regard to these aspects of patient care.

**Methods:**

A telephone survey of family physicians in the Jerusalem, Israel, district using a standard questionnaire.

**Results:**

74 family physicians responded to the survey. The response rate was 42 % and the cooperation rate was 66 %. Most of the respondents, (64 %), reported knowing that the PDM delegation facility exists, though only 24 % claimed to have suggested to their patients that they consider this option. Approximately three-quarters, (78 %), treat patients with whom they discussed other aspects of severe disease, disability or EOL. None of the physicians working predominantly with religiously observant groups reported suggesting PDM delegation.

**Conclusions:**

There is an apparent gap between family physician knowledge and their performance to empower the persistence of patient autonomy, should communication ability cease. System-wide interventions to increase EOL communication skills, starting at medical school and henceforth, are necessary in order to promote better EOL care and meaningful resource use.

## Background

During the latter part of the 20th century, the concept of patient autonomy surfaced, evolved and diffused to all aspects of healthcare. Associated challenges include circumstances in which patients are unable to communicate and actively divulge their thoughts, preferences and wishes; nor can healthcare providers elicit them.

The tools available in these circumstances include knowledge of the patient preferences and end-of-life (EOL) instructions prior to communication incapacity through validated documentation (paper, film or other media). However, applying these instructions can be challenging and the interpreters’ ethical and cultural standpoints affect decision-making. Legal facilities exist to designate court-appointed proxy decision-makers. In these circumstances, the proxy decision- makers (PDM) might not know what the patient would have wanted and which ideals and preferences should guide them.

A third, middle road possibility, is that the patients, when they have communication capacity, designate a PDM, either with or without power of attorney, identifying the people they entrust with the responsibility to fulfil their autonomy if communication capabilities diminish. This option allows patients to discuss their preferences and morals with their designated PDM. In cases of diminished communication capacity, The PDM would have already been introduced to the critical issues, know of any explicit preferences and have the flexibility and spirit to manage various clinical scenarios that arise. The healthcare staff can assist the PDM in these deliberations, knowing that they are doing their best to fulfill the patient’s autonomy.

Many patients might prefer to discuss these matters during their better health periods in the clinic rather than during illness [1]. Family/primary-care physicians seem to be suitable to assist patients with EOL instructions and designation of a PDM because they are sensitive and close to the patients in their cultural and genealogical surroundings. They are also able to revisit these topics over time, and allow maturation of the issues and dilemmas. Physician initiative is important and the troubling parts of the discussion seem not to discourage patients, but rather more, the physicians [2]. Patients who are in better contact with their primary-care physicians have been shown to be more aware of PDM designation possibilities [3]. The SUPPORT project (Study to Understand Prognoses and Preferences for Outcomes and Risks of Treatments) promoted communication focused on understanding patient beliefs and preferences regarding various medical procedures, prior to their need [4]. Yet most of the evidence on patient EOL instructions and PDM designation comes from acute care settings and patients who are ill, rather than stable and relatively healthy [5].

We sought to assess the willingness of primary-care family physicians to assist patients and promote designation of a PDM and sought to identify perceived obstacles in order to establish a framework for interventions and improvement.

## Methods

A cross-sectional telephone survey of family physicians using a standardized questionnaire. We approached all 176 primary care physicians registered in the Jerusalem district of Clalit health services (CHS – the largest governmentally funded healthcare insurer and provider), treating approximately 430,000 enrollees.

The questionnaire presented respondents with phrases assessing physician actual practices regarding patient PDM appointment, phrases about physician concerns regarding the topic, and phrases assessing to which patients physicians should offer PDM appointment and discussion of the topic (detailed in the results tables). The respondents were asked to mark either agreement or disagreement with each of the phrases. We collected additional information about the served population characteristics and physician experience. The form was pilot tested, assessed and improved with several senior geriatric and family physicians. Additionally, a forum of family physicians filled the form and then, in open discussion, reviewed the questionnaire and proposed revisions until content and clarity reached saturation.

We contacted all clinics; there were several attempts to recruit all registered physicians. In order to ensure participant anonymity, we discarded the list of participating physicians at the end of the survey period. There was no participant identification coded otherwise. The CHS ethics IRB approved the study, allowing data collection and inclusion only from physicians agreeing to participate. Using the chi-square test, we compared groups of doctors who reported having offered PDM appointment to those who did not. Pilot questionnaires were not included in physician survey results.

## Results

In the Jerusalem district survey (June 2012), there were 176 registered physicians of whom 63 were not available (vacation, leave etc.). Of the remaining 113 physicians, 33 were not interested in participating; six did not find the time to answer the telephone questionnaire and 74 physicians participated. Thus, the respondents constituted 42 % of the original sample (the response rate) and 66 % of those available (the cooperation rate). Young physicians (under 45) comprised 12 and 23 % were older than 60. The mean (SD) age of all the respondents was 53 (7), with 26 (7.5) years in the profession and 14 (8) years working in the clinic they were approached at. Female physicians constituted 40 % of the participants.

Most of the respondents (90 %) worked in urban clinics. Almost two thirds of participants (63 %) served populations that were mostly Jewish (63 %) and 24 % served populations that were mostly Muslim Arab. Physicians classified the communities they work at to be religious (27 %), observant/traditional (63 %) and secular (9 %).

Most physicians: discussed EOL topics with their patients, reported knowledge of the legal option to identify a PDM; did not discuss PDM delegation with their patients (Table 1). Among physicians who reported having discussed with their patients the topic of PDM appointment, 67 % reported that patients opted to do so. We found that 70 % of the physicians, who did not discuss/offer PDM delegation, stated they wanted to do so. Most physicians reported having received communication skills training. Participants most often stated that the professional most appropriate to discuss the topic is the family physicians (48 %). Other options were non-medical professionals from community services (25 %), hospital doctors (12 %) and in-hospital non-medical professionals (9 %).

**Table 1:** Physician responses regarding proxy decision-maker (PDM) delegation (*n* = 74)

Aware of patient option of PDM delegation	48 (64 %)
In preceding year, had discussions with patients on topic of death/ severe disease/ significant disabilities/ dependence on others.	58 (78 %)
In the past year, offered patients to delegate PDM.	18 (24 %)
Undergone patient-physician communication workshops and training.	55 (74 %)
Feel they had not received adequate training to discuss PDM delegation.	38 (51 %)
Feel that the time available in clinic visit is insufficient for discussion of the topic.	45 (60 %)
Feel close to the patients in clinic.	72 (97 %)

We asked physicians about various impediments to PDM delegation discussions and grouped these into four categories: potential medical hazards caused by raising the topic; potential communication and relationship hazards; sense of futility; physician uneasiness (Table 2). The most common agreement was that patients might change their minds regarding EOL instructions.

**Table 2:** Agreement with phrases which might impede discussion of PDM

Fear of harm to patient	Discussing PDM has the potential to depress the patient	39 (53 %)
	Discussing PDM can lead to medical deterioration	13 (18 %)
Fear of relationship disruption	Discussing PDM could harm the doctor-patient relationship	23 (31 %)
	Delegation of PDM can lead to familial disputes	37 (50 %)
Sense of futility	Patients are not interested in discussing delegation of PDM	28 (38 %)
	Patient do not have the capacity to appreciate the complexity of their medical condition and are not fit for discussing and deciding on this topic	21 (28 %)
	Patients might change their mind regarding EOL instruction, with time and given varying situations.	69 (93 %)
Physician uneasiness	I do not feel comfortable to discuss the topic	21 (28 %)
	I feel burdened by the topic	46 (63 %)

The majority of participants (91 %) thought that discussing PDM designation was relevant when patients were suffering from advanced / severe morbidities such as cancer, heart failure and renal failure. Far less (47 %) thought it appropriate to discuss this topic with patients about to undergo surgery / anesthesia. Raising the matter with all elderly patients was thought appropriate only among 24 % of the respondents.

Most respondents (84 %) did not agree that PDM appointment is useless; and a similarly large percentage agreed (80 %) that difficult situations should only be dealt with when they occur and not pre-emptively. About half (57 %) stated they were interested in appointing a PDM for themselves in case they are incapable of communication and that they would be willing to act as a PDM for a family member or a friend.

When we compared physicians who did and did not discuss PDM delegation (Table 3), we found that those who did not, more often, reported that such discussions could hurt their patients and cause dispute. They also reported not feeling comfortable raising the topic. None of the physicians working with religiously observant communities reported suggesting PDM delegation to their patients. Most physicians reporting PDM discussions were between 45 and 60 years old (84 %). In this age group 31 % of physician reported PDM discussion with their patients whereas in younger and older physician age groups 11 % reported doing so.

**Table 3:** Bivariate analysis: FPs who discussed PDM vs. those who did not

	Yes *N* = 18	No *N* = 56	*p*-value
Patients are not interested	5 (28 %)	23 (41 %)	0.4
Cause family dispute	5 (28 %)	32 (57 %)	0.05
Depress the patient	4 (22 %)	35 (62 %)	0.006
Compromise medical status	0	13 (23 %)	0.03
Patients cannot perceive the consequences of their decisions	3 (17 %)	18 (32 %)	0.24
Patients might change their minds	16 (89 %)	53 (95 %)	0.59
I do not feel comfortable to discuss the topic	1 (6 %)	20 (36 %)	0.02
I feel burdened by the topic	12 (66 %)	34 (60 %)	0.7
Harm doctor/patient relationship	3 (16 %)	20 (36 %)	0.16
I do not believe discussions are of any good	1 (6 %)	11 (20 %)	0.33
There is no reason to discuss this issue before anything really happens	0	15 (27 %)	0.02
Should discuss with all elderly patients	5 (28 %)	13 (23 %)	0.76
Should discuss with patients about to undergo surgery	10 (56 %)	25 (46 %)	0.6
Should discuss with patients suffering life-shortening diseases	16 (89 %)	52 (93 %)	0.63

## Discussion

The most striking results in this study are the discrepancy between physicians’ knowledge of the PDM option and the limited use and discussion of this option with patients. Most physicians indicated that they had patients with significant co-morbidities/disabilities or life threatening conditions, which enhances the incongruity. Another striking result was the widespread agreement with the statement that patients in practice change their minds regarding EOL instructions.

Many of the respondents stated that they did not feel they had received the necessary training to discuss delegation of PDM with their patients. All physicians are medical school graduates and most had participated in formal training in patient-physicians communication skills as part of their resident training. The low rate of PDM delegation discussions is not an error in system processes; rather, it reflects a situation in which many medical institutions do not prioritize the issue. Our results suggest that teaching and training institutions as well as administrative and regulatory institutions, do not value the issue to a notable extent that generates training, skill and action and appropriate service.

In all societies, EOL is an arena in which ethical and legal schools of thought clash. Yet, we should note that unlike other EOL instruction facilities, delegation of PDM is the least controversial, allowing both pro-life idealists as well as their opposites, alike, to maximize the potential to fulfil their autonomy, if they have the misfortune to lack communication capacity. PDM appointment, more than other options, allows the greatest future flexibility and is far less stringent than other EOL empowering / instruction arrangements. Thus, the physicians’ expressed fear of changes in patient priorities would be best served by PDM appointment, rather than specific instruction.

Change and improvement in physician initiatives and promotion of PDM appointment could be sought in basic medical education. The medical curriculum is already congested with increasing requirements to add material and training to the classical core. EOL care, including PDM delegation, has to compete with other topics on resource allocation in the medical schools.

Previous surveys have shown that 19 to 55 % of severely morbid patients are approached to discuss end-of-life care topics [1, 6, 7]. Interventions to improve the prevalence of EOL instructions in outpatient clinics have been shown to increase these from 24 to 38 % [8]. Similar attempts in hospitalized patients increased the prevalence of EOL instructions from as low as 0 % to as high as 36 % [9, 10]. Unsuccessful interventions have also been reported [11].

One of the limiting factors of EOL instructions and designation of PDM is lack of physician initiative [2, 3]. Some physicians believe that such discussions can harm patients [12]. Other physicians are afraid that such discussions could harm their relationship with their patients [4]. This contrasts with studies which have shown that shared formation of EOL instructions strengthens patient-doctor relationships and that patients do wish to discuss these topics, even if stress and anxiety ensue [1, 3].

The Israeli Law for the Dying Patient is an attempt to balance between the values of patient’s autonomy and sanctity of life [13]. The law establishes procedures for appointing proxy decision-makers and leaving advance medical directives. Every 5 years, a national registry reminds, those who have filed advance directives to check whether they have changed their minds. The registry serves as a source of information whenever an incompetent patient is admitted to a hospital and it is unknown whether there is an advance directive. Testimony about patient’s wishes by family or friends is also valid. While the law was enacted a decade ago, physicians still have poor knowledge of it [14] and implementation has been slow [15]. Despite legislation aimed toward expansion of individual autonomy, current policy promotes court-appointed guardianship for all patients with dementia rather than eliciting individual preferences when this is still an option [16]. There is a recognized need for increased awareness among the public and training health care providers to conduct timely conversations about preferences for end-of-life care [14–16]. PDM are people identified by patients as being the ability to deliberate and communicate the patients’ autonomy. This should not be confused with legal representatives charged to be acting guardians of the patients.

Another driver of change could be quality measures. EOL care is a very delicate personal, ethical, cultural and political topic, to say the least [17]. Yet, PDM has the potential to receive wide public acceptance, among various belief holders. As a quality measure, it would reflect far more than procedural appointment, and would probably promote patient-doctor-system coordination, communication and confidence. However, quality measures might lead to gaming strategies, draining the topic from its content and lead to a system aggressively seeking to discuss the matter only for the sake of appearance and rating.

Participant selection bias restrains the external validity of our findings and conclusions. The ethical approval we received allowed us to include information about only those physicians who agreed to participate in the study. Therefore, we were not in a position to gather data about physicians in the Jerusalem district who work in CHS and cannot truly account to the extent of selection in that group. Comparing the age and sex composition of our participants, 44 % of randomly selected physicians in a national survey of primary care physicians were female [18] and 45 % of all registered community working physicians in a 2012 census were female [19]; similar to our group composition. However, the age distribution of our participants differs substantially from that of the aforementioned reports: In the random survey of primary care physicians approximately 26 % were under 45 and 19 % were older than 60 [18]; in the national physician census 22 % were under 45 and 11 % were older than 65 [19]. Our participants, in that respect are somewhat different: 12 % were younger than 45, and 23 % were older than 60. The basic demographic comparisons do not disclose any clear bias that might have been introduced in this current report; however, they certainly suggest that the generalizability of our findings is not straightforward.

This report should be cautiously interpreted. The Jerusalem district is unique as the various religious inclinations might be more intense due to the association with the holy city and sacred history. Indeed, physicians caring for mostly observant communities did not report PDM delegation discussions. Among physicians and patients of all religions, tension might be raised between opposing standards brought by religious and spiritual influences. Additionally, the study gathered only physicians self-reported actions and thought. There is no systematic standard to document EOL associated actions and, additionally, retrieve data regarding the actual behavior of family physicians in Israel.

Finally, in an era of austerity and financial constraints, there is growing appreciation of the resource consumption that occurs during the end of life, often with a sense of significant futility. In many instances, the medical imperative, when no legal avenues exist for withholding treatment, is to provide any and all treatment modalities available, as dictated by the clinical entities the patient presents with. Opportunities to communicate with people chosen and empowered by the patient to be the optimal agents of his will can enable treatment in accordance with his wishes or guided by his ideals. This could result in less costly, more humane and palliative care within a legal framework and provide confident value when resources are used, knowing that this fulfills patient preferences, thus adding to their worth, perhaps decreased moral stress [20].

## Conclusions

Family physicians in Israel are not, as of yet, potent facilitators of PDM delegation. It seems that this topic has not been the focus of the medical establishment’s components, from medical school, continuing medical education or quality monitoring. Specific EOL directed teaching, training, skill maintenance and monitoring could serve patients and society by promoting autonomy fulfillment and meaningful care, whether palliative and invasive, passive or active.

## Abbreviations

EOL: End-of-life
PDM: Proxy decision-makers
